# Genome-wide linkage scan for loci of musical aptitude in Finnish families: evidence for a major locus at 4q22

**DOI:** 10.1136/jmg.2007.056366

**Published:** 2008-04-18

**Authors:** K Pulli, K Karma, R Norio, P Sistonen, H H H Göring, I Järvelä

**Affiliations:** 1 Department of Medical Genetics, University of Helsinki, Helsinki, Finland; 2 Department of Music Education, Sibelius Academy, Helsinki, Finland; 3 Department of Medical Genetics, Family Federation of Finland, Helsinki, Finland; 4 Red Cross Finland Blood Service, Helsinki, Finland; 5 Department of Genetics, Southwest Foundation for Biomedical Research, San Antonio, USA; 6 Laboratory of Molecular Genetics, Helsinki University Hospital, Helsinki, Finland

## Abstract

**Background:**

Music perception and performance are comprehensive human cognitive functions and thus provide an excellent model system for studying human behaviour and brain function. However, the molecules involved in mediating music perception and performance are so far uncharacterised.

**Objective:**

To unravel the biological background of music perception, using molecular and statistical genetic approaches.

**Methods:** 15 Finnish multigenerational families (with a total of 234 family members) were recruited via a nationwide search. The phenotype of all family members was determined using three tests used in defining musical aptitude: a test for auditory structuring ability (Karma Music test; KMT) commonly used in Finland, and the Seashore pitch and time discrimination subtests (SP and ST respectively) used internationally. We calculated heritabilities and performed a genome-wide variance components-based linkage scan using genotype data for 1113 microsatellite markers.

**Results:**

The heritability estimates were 42% for KMT, 57% for SP, 21% for ST and 48% for the combined music test scores. Significant evidence of linkage was obtained on chromosome 4q22 (LOD 3.33) and suggestive evidence of linkage at 8q13-21 (LOD 2.29) with the combined music test scores, using variance component linkage analyses. The major contribution of the 4q22 locus was obtained for the KMT (LOD 2.91). Interestingly, a positive LOD score of 1.69 was shown at 18q, a region previously linked to dyslexia (DYX6) using combined music test scores.

**Conclusion:**

Our results show that there is a genetic contribution to musical aptitude that is likely to be regulated by several predisposing genes or variants.

Music is an ancient and universal feature across all human societies. The ability to appreciate music requires no explicit training. The universality of musical behaviour and validity of common rules such as use of octave-based scale systems and preference for consonance over dissonance in nearly all types of music can be seen as evidence for innateness. Rules have arisen independently in isolated cultures, and some of them also apply to the music perception of non-human species. This implies that these rules have their basis in brain organisation rather than in culture.^1^

Observations on fetuses and infants have revealed that basic auditory abilities, such as pitch discrimination and also more complex capabilities such as melody recognition, are already present in the early stages of development.^2^ It has been suggested that this is partly due to prenatal exposure to musical stimuli. However, adults’ abilities to perceive music is somewhat dependent on culture, whereas infants seem to possess a more generalised capability, which obeys the aforementioned universal rules of music.^2^ This implies the existence of an innate ability, which later in life can be modified by environmental effects. A fundamental question is whether, or at what level, this ability is genetically determined.

Neuroimaging and neurophysiological studies have shown that listening to and/or playing music has multiple effects on brain structure and function, suggesting a biological effect. Neurophysiological studies have shown that musical stimuli activate specific areas of the brain.^3^ ^4^ Active training and practising of music has been shown to enlarge some areas of the brain,^5^ and the role of genetic predisposition to the morphological and neurophysiological changes has also been discussed.^4^ In investigations using positron emission tomography (PET), listening to music has been reported to cause physiological changes in cerebral blood flow, cardiovascular and muscle function.^6^ However, the molecules mediating these responses remain uncharacterised.

It has been observed that professional musicianship clusters in families. How much this aggregation is due to genetic or environmental factors has been the subject of debate.^7^ ^8^ It has been shown that a genetic component exists in exceptional phenotypes of musical aptitude: absolute pitch (the ability to name isolated pitches without a reference pitch^9^) and more recently in congenital amusia (tone deafness).^10^ Moreover, there has been reported a difference between monozygotic and dizygotic twin pairs in the ability to recognise anomalous pitches in simple popular melodies using the Distorted Tunes Test.^11^ To our knowledge, no gene loci or genes for any of these traits have been identified.

Musical ability varies between individuals, and seems to be expressed at the population level in such a way that both extremes (extremely capable or incapable individuals) are rare, and hence most individuals express moderate ability.^12^ This is a typical feature of a complex trait influenced by several underlying genes, environmental factors and their interactions. We hypothesised that musical aptitude is an innate cognitive ability that is partly under genetic regulation and serves as a basis for musical expertise in a favourable environment. To investigate this hypothesis, we recruited families and performed heritability analysis and genome-wide linkage analysis.

## METHODS

The ethics committee of Helsinki University Central Hospital approved the study, and informed consent was obtained from all participants.

### Subjects and phenotype assessment

In total, 234 people from 15 families of Finnish origin with some professional musicians and/or active amateurs were recruited for the study via a nationwide search by sending information leaflets or letters to the families whose members had studied or were studying at Sibelius Academy or music institutes in Finland (supplementary fig 1 online). The family members who first contacted us knew that there were some musicians or active amateurs in their families and informed their families about the study. Among the professional musicians, the two main groups were pianists and violinists. The amateurs were most commonly playing the piano or singing in a choir. The phenotypes of all family members were defined by three tests for musical aptitude: an auditory structuring ability test (the Karma Music test, KMT) designed by one of the authors,^13^ and the Seashore pitch and time discrimination subtests (SP and ST respectively).^14^ The KMT has been successfully used for 30 years in Finnish primary schools and music institutes a selection test for students to play an instrument. The KMT is devised to measure auditory structuring in a way that minimises the effects of training and/or culture.^15^ It uses small, abstract sound patterns that are repeated to form hierarchic structures. The subject’s task is to detect structural changes in these patterns, ie, changes in the order or number of the tones (an example of three items is enclosed in the supplementary material online). In a study that used the Mismatch Negativity (MMN) component of event related brain potentials, persons with high scores in the auditory structuring test were compared with those who had low score. There was a difference when the stimuli were sound patterns but not when mere pitches were used. This supports the validity of the test as a measure of auditory structuring rather than sensory differentiation.^16^ In contrast, the SP and ST subtests consist of pair-wise comparisons of the physical properties of sound and are used to measure simple sensory capacities, such as ability to detect small differences in tone pitch or length. Testing was performed as a group test that lasted about 1 hour. Groups contained members from one family or from multiple families in each “session”. A total of 224 family members (105 males and 119 females) completed all tests and thus were included in further analyses.

**Figure 1:**
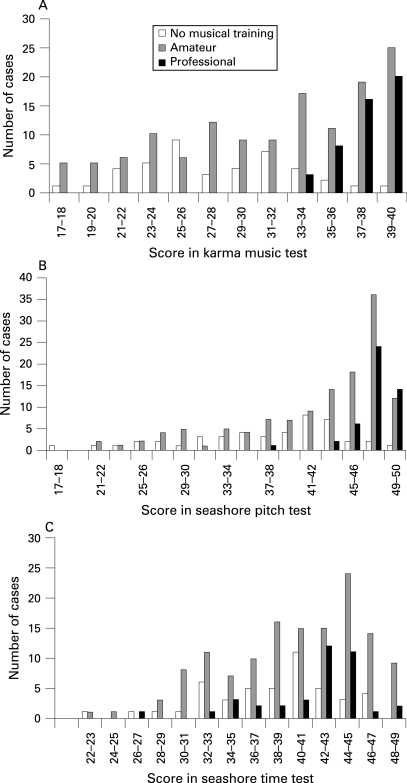
Scores in the three musical aptitude tests clustered by amount of training and professional status in music (n = 224). Because of the way the sample was collected there are more high scores than in the population in general. This causes some ceiling effects, but making the test difficult for professional musicians would probably make it too difficult for the average subject and cause guessing, which lowers the reliability of the measurement.

All of the tests used a continuous scoring scale (for KMT, 0–40; for SP and ST, 0–50), with one point obtained for each correct answer. The reliabilities (α coefficients) of the tests were: KMT 0.88, SP 0.91 and ST 0.78. Calculating reliabilities is a standard procedure within the behavioural sciences. This index is an estimate of the share of systematic, non-random variance in measurements (maximum = 1). The correlations between the three tests were: KMT/SP 0.64, KMT/ST 0.41 and SP/ST 0.41. These results show that the tests are reliable and only partially overlapping.

The results of the three music tests were given to the participants if they were interested. Instructions for carrying out the tests are available on request.

### Phenotype processing

To get a rough idea of the age where musical aptitude stabilises, we analysed the relationship between the musical aptitude test scores and age in three groups (ANOVA, table 1). In all tests the results of the youngest age group were significantly lower than those of the older groups. The group of 9–11-year-olds scored on the same level as those aged >12 years (p = 0.831 for KMT; p = 0.759 for SP and p = 0.362 for ST). The result is in agreement with the previous studies.^15^ ^17^ These data suggest that the maturation of the brain for musical aptitude occurs relatively early.

**Table 1:** Musical aptitude test means by three age groups

	KMT	SP	ST
Age group			
<9 years (n = 7)	23.0 (4.6)	35.6 (9.2)	33.4 (3.2)
9–11 years (n = 13)	32.2 (5.7)	43.2 (6.5)	39.1 (5.2)
>11 years (n = 205)	32.5 (6.4)	42.6 (7.0)	40.6 (5.5)
p Value*			
<9 or 9–11 years	0.002	0.045	0.001
<9 or >11 years	0.000	0.011	0.001
9–11 or >11 years	0.837	0.770	0.325

*Between means.

Data are means (SD) except for p values (n = 224).

ANOVA was used for analysis.

We also characterised the relationship between the music test scores and training status (fig 1). A questionnaire was used for rough categorisation of training into three classes: professionals, amateurs and people with no music training at all. Of the 224 family members, 47 were professionals, 135 were amateurs and 42 were people who were not musically trained. When training was quantified according to the above classes as 0, 1, and 2, it correlated with the tests as follows: KMT/training 0.49, SP/training 0.45, ST/training 0.23. Although there was a clear connection between musical training and the scores, the source of this relationship was not revealed by the scores. Training may have driven performance and/or performance was driven by selection because substantial innate musical aptitude is necessary in order to become a successful professional musician. Three lines of evidence support the view that KMT is not sensitive to training effects. First, musical training is not a necessary condition for a high score. We have previously shown that some subjects score at professional level without any musical training^15^ and this was also found in the present study. Second, with applicants for lower level music instruction, KMT had low correlations with earlier music training and the correlations with other measures of musical ability (sum of playing, singing and an echo test) were higher in all age groups.^15^ Third, in the aforementioned study by Tervaniemi *et al*,^17^ the effects in event-related brain potentials could be explained by the KMT but not by the musical training of the subjects. The data in KMT and SP (fig 1) could be interpreted to indicate a selection effect; it was impossible to become a professional musician without good auditory structuring and pitch differentiation ability. In contrast, there were some professionals with scores lower than the whole sample mean in the Seashore time test. This may have been partly a consequence of the rather “unmusical” nature of the test. Absolute lengths of the tones were compared instead of onset distances, which probably would be a much more “musical” task type.

We conducted statistical genetic analyses (heritability and VC linkage analysis) on the separate scores of the three music tests and also on a combined music test score, as we hypothesised that there would be a common denominator for the three tests. In preparation, we made the following adjustments and transformations. A few people (8 for KMT, 6 for SP, and 2 for ST) scored below the expected value for random guessing of answers (50% of answers correct), and their scores results were raised to these values, under the assumption that any differences in scores below the guessing limit are solely due to chance, rather than being a reflection of differences in ability. The combined phenotype was subsequently computed as the sum of the z-scores of the three individual test results. We summed the z-scores (ie, values after subtraction of the mean and division by the standard deviation) to weight all three tests equally. This would not be case if we had summed the raw scores, as the tests have slightly different ranges. An exact inverse normal transformation was subsequently used on all phenotypes (KMT, SP, ST, and combined) to ensure a normal distribution. Sex, age, and age^2^ and their interactions were included as covariates in all analyses, using a linear regression model for the phenotypic mean.

We performed a simulation study to estimate the power to detect linkage in our pedigree sample. In agreement with the small sample size, the study had only modest power to detect linkage. Assuming a heritability of 48% (identical to our estimate for the combined phenotype), we estimated that the study had 50% power to detect linkage with a LOD score of 3 for a locus explaining ∼55% of the phenotypic variance.

### Genotyping

Family members >12 years of age were allowed to give a DNA sample for molecular genetic analyses. After obtaining informed consent, venous blood samples were collected from the participants and their DNA was extracted using standard procedures. Altogether, genotype data was obtained for 205 participants. Genotyping was conducted with 1113 microsatellite markers with an average marker density of 3.3 cM. The genetic locations of the genotyped markers were based on the genetic map developed by deCODE genetics.^18^

### Statistical genetic analyses

Genotyping errors were “cleaned” using the multipoint approach implemented in the software package SimWalk2, eliminating inconsistent genotypes and those that appear unlikely to be correct.^19^ ^20^ Marker allele frequencies were estimated by maximum likelihood,^21^ and allele sharing probabilities were computed in at multipoint fashion using the approximate Monte Carlo Markov chain approach implemented in the software package LOKI.^22^ For heritability and linkage analyses,^23^ variance component (VC) analysis was conducted using the SOLAR software package with an additive model of allelic effects.^24^ All statistical genetic analyses were based on 224 participants with complete phenotype and covariate data.

## RESULTS

### Variance component heritability and linkage analyses

The heritability estimates are shown in table 2. All examined phenotypes showed a substantial heritability, ie genetic differences between individuals explain a substantial proportion of the phenotypic variance. The highest heritability was obtained with SP and the lowest with ST.

**Table 2:** Heritability estimates of the test points

Test	h^2^ (%)	p Value
Karma Music Test	42	3×10^−5^
Seashore pitch	57	1×10^−7^
Seashore time	21	0.03
Combined	48	1×10^−5^

**Figure 2:**
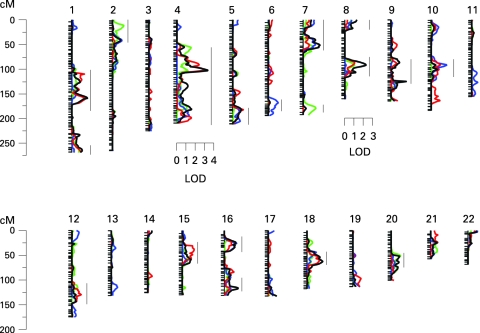
The genome-wide LOD score plot of musical aptitude using KMT (red), SP (blue), ST (green), and the combined phenotype (black). The marker locations were based on the deCODE genetic map. Chromosomal locations with a LOD scores >1 with any of the traits (KMT, SP, ST and/or combined) are indicated by a vertical line.

**Figure 3:**
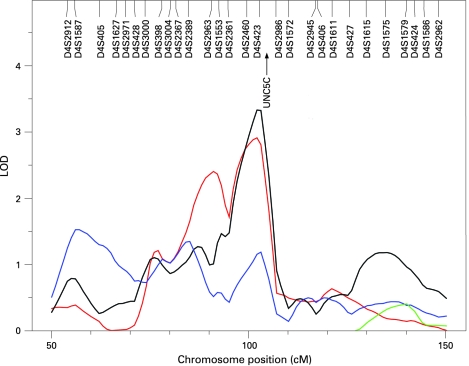
Linkage of musical aptitude defined by KMT (red), SP (blue) and ST (green) and combined phenotype (black) to chromosome 4q.

**Figure 4:**
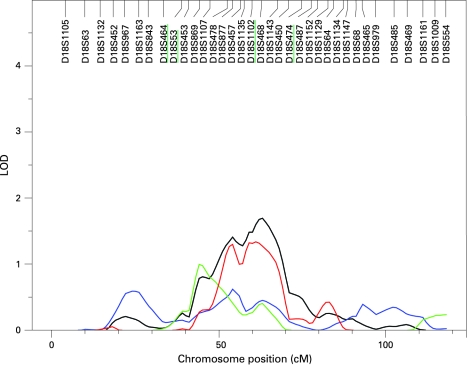
Linkage of musical aptitude defined by KMT (red), SP (blue) and ST (green) and combined phenotype (black) to the DYX6 locus on chromosome 18q. The microsatellite markers with the highest linkage values of the DYX6 locus are underlined.

The results of the multipoint linkage analysis are illustrated in fig 2. As expected, given that we used an inverse normal transformation and that the covariates had only moderate effects, the residual kurtosis after accounting for the effects of covariates was not marked (0.364 for KMT, 0.493 for SP, −0.079 for ST and 0.461 for combined phenotype), and thus the results of the VC linkage analysis are robust and can be interpreted in the standard way, with a LOD score ⩾3 indicating significant evidence of linkage.^25^ Significant evidence for linkage (LOD 3.33) was found using the summarised test scores on chromosome 4q22, near markers D4S423 and D4S2460 (genetic map location 102 cM) (fig 3). This result appears to be driven mainly by KMT, which yielded a LOD score of 2.91 (102 cM), and somewhat by ST, which gave a LOD score of 1.18 at 103 cM. However, there was no evidence of linkage to this region with SP. Suggestive evidence of linkage was obtained on chromosome 8q (LOD = 2.29 at genetic map location 92 cM) with the combined scores. The highest linkage peak for SP was a LOD score of 1.67 at 92 cM on chromosome 10. Interestingly, the combined scores yielded a LOD score of 1.69 on chromosome 18q (at 63 cM), which overlaps with the DYX6 locus previously shown to be associated with dyslexia^26^ (fig 4). These findings show that the test results correlate only partially and are in agreement with the results shown above. In addition, there were 15 chromosomal regions with a LOD score >1 (fig 2).

## DISCUSSION

To unravel the biological basis for musical aptitude, we performed genome-wide linkage analysis using microsatellite markers spanning the genome at ∼3 cM density and were able to show significant evidence for linkage at 4q22. In silico analysis revealed of a total of 50 genes between markers D4S2986 and D4S2361 (http://www.ncbi.nlm.nih.gov). In the highest peak region, between markers D4S423 and D4S2986, an interesting candidate gene appeared: the netrin receptor UNC5C precursor interacts with netrins that direct axon extension and cell migration during neural development. Netrins interact with robo family receptors.^27^ Interestingly, *ROBO1* is known to be a candidate gene for dyslexia.^28^ The interaction of netrin receptors and robo family receptors raises a question about the common molecular background of music and language faculties. Furthermore, mutations in the UNC5C homologue can cause cerebellar defects in rodents.^29^ Cerebellar damage is shown to cause deficits in pitch and especially time processing in humans.^30^ Another member of the netrin receptor family, UNC5B, has been mapped to 10q22.1 where we found a LOD score of 1.67.

On chromosome 8q13-q21, where we obtained suggestive evidence for linkage with the combined phenotype, there is a member of the transient receptor potential family of ion channels, *TRPA1*, which has been suggested to be a candidate for the mechanosensitive transduction channels of hair cells in vertebrate inner ear. The structure and mechanism of these channels that mediate hearing and balance has been predicted, but their molecular function remains unknown. It has been suggested that TRPA1 could be a non-essential subunit of transduction channel.^31^ This makes it an interesting candidate gene for musical aptitude. Because of lower selection pressure, it might be prone to mutations and thus developed different forms, which could account for the slight variation between individuals’ sound perceptions.

There has been discussion about the common origin of music and language faculties.^1^ Dyslexia is a common constitutional syndrome that has a strong genetic component. A total of nine chromosomal loci for dyslexia (*DYX1–9*) and several putative genes have been assigned in the human genome.^27^ We have previously suggested that an important predictor of dyslexia, phonological awareness, can be understood as poor auditory structuring ability applied to language and have shown that KMT significantly predicts dyslexia.^12^ Strong evidence for linkage at the DYX6 locus has been obtained with several component phenotypes of dyslexia, one of which is phoneme awareness,^31^ a subset of phonological awareness. The putative common locus for musical aptitude and dyslexia at 18q refers to a common evolutionary origin of music and language faculties.

Our study is based on multigenerational families, which is expected to reduce the number of genes or alleles associated with musical aptitude compared with case–control studies. The study was performed in families originating from an isolated population of Finland, where founder effect and genetic drift have shown to restrict the number of alleles in human diseases.^32^ Common family environment and lifestyle should further reduce the number of confounding factors affecting the results. Nonetheless, the most serious limitation of this study is the relatively small sample size, leading to imprecise estimates of heritability and a low power to detect linkage, except for loci with very large effect sizes. Given the small sample size, we did not attempt to perform bivariate or trivariate linkage analyses with the individual music test scores, because this would necessitate additional parameters or degrees of freedom that probably would severely reduce power. Instead, to localise shared genes influencing these traits while avoiding model complexity, we combined the individual music tests scores into an overall score in an ad hoc manner, weighting each of the three separate scores equally. For a study of this size, this procedure is likely to lead to higher power. Another complication is that there were more high scores than low scores in the families due to selection bias. This might reflect a strong genetic component of musical aptitude, but the effect of a favourable environment cannot be excluded. Correcting for ascertainment is notoriously difficult (except in specific cases such as “single ascertainment”), and we thus did not attempt to do so. This is conservative, because not correcting for ascertainment is not expected to increase the false positive rate, but rather lead to a reduction in power.

Our study represents the first systematic molecular genetic study to attempt identification of candidate genes and genetic (biological) variants associated with musical aptitude. The results of our study suggest that musical aptitude is an innate ability that is associated with several predisposing genetic variants. The current work represents a starting point for further mapping, isolation and characterisation of genes that predispose to musical aptitude. The identification of genes or genetic variants involved in mediating music perception and performance should offer new tools to understand the role of music in human brain function and human evolution, and its relationship with language faculty.

**Key points**

- We showed that three tests of musical aptitude, an auditory structuring ability test (Karma Music test; KMT), Seashore test for pitch (SP) and for time (ST) showed substantial heritability in 15 Finnish families
- Significant evidence of linkage was obtained for chromosome 4q22 (LOD 3.33) and suggestive evidence of linkage for 8q13-21 (LOD 2.29), with the combined music test scores using variance component (VC) linkage analyses in the Finnish families
- Our results show that there is a genetic contribution to musical aptitude that is likely to be regulated by several predisposing genes/variants.
